# Supplementary material for: Microbial community composition and function in an urban waterway with combined sewer overflows before and after implementation of a stormwater storage pipe
Source: PeerJ. 2023 Jan 12;11:e14684. doi: 10.7717/peerj.14684 (PMC9840855; doi:10.7717/peerj.14684)
Supplement: Supplemental Information 1 [file peerj-11-14684-s001.docx]

**Supplemental information**

Microbial community composition and function in an urban waterway with combined sewer overflow before and after implementation of a stormwater storage pipe

Kazuaki Matsui^a)^ *, Takeshi Miki^b)^

^a^ Department of Civil and Environmental Engineering, Kindai University, 3-4-1Kowakae, Higashiosaka, Osaka 577-8502, Japan.

^b^ Faculty of Advanced Science and Technology, Ryukoku University, 1-5 Yokotani, Seta Oe-cho, Otsu, Shiga 520-2194, Japan.

* Corresponding author: kmatsui@civileng.kindai.ac.jp

TABLE OF CONTENTS

| **Table S1** | Summary of the rain fall events on each sampling period | S2 |
| --- | --- | --- |
| **Table S2** | Sampling time information | S3 |
| **Table S3** | Information for DNA recovering efficiency determination | S4 |
| **Table S4** | Primers used for screening of tetracycline resistant bacteria | S5 |
| **Table S5** | Primers and probes used in quantitative PCR assays | S6 |
| **Table S6** | Statistics tables for Fig. 2 | S7 |
| **Table S7** | Statistics tables for Fig. S3 | S8-9 |
| **Table S8** | Identified tetracycline resistant environmental isolates carrying *tet* genes | S10 |
| **Fig. S1** | Standard curve of *tet*(A), *tet*(B), and *tet*(M) for qPCR assay. | S11 |
| **Fig. S2** | Depicted image of stagnant water in combined sewer overflow structure | S12 |
| **Fig. S3** | Principal Coordinate Analysis (PCoA) of physicochemical and ecological variables from 3 stations sampled in 3 phases. | S13 |
| **Fig. S4** | The box-and-whisker plots *tet*(A), *tet*(B), and *tet*(M) genes at stations A, B and C in 3 different phases | S14 |

**Table S1** Summary of the rain fall events on each sampling period

**Table S2** Sampling time information

**Table S3** DNA recovery efficiencies of four procedures.

***Determination of DNA recovery efficiency***

DNA recovery efficiency was determined using *E.coli* JM109, which has seven 16S rRNA gene copies (Stoddard *et al.* 2015). 16S rRNA gene copies were normalized to the initial cell number.

*E. coli* was cultured in Luria–Bertani medium at 37℃ for 18 h with vigorous shaking and stored on ice. *E. coli* cells were enumerated under an epifluorescence microscope by SYBR Green I staining (Honjo *et al.* 2006). An *E. coli* culture (100 μL; 1.3 × 10^6^ cells/µL) was trapped on a 0.2-µm nucleopore polycarbonate filter (47 mm diameter; Advantec, Japan) and transferred into a tube containing ISOIL for bead-beating (Nippon Gene, Japan) to evaluate DNA recovery. For DNA extraction without filter trapping, 100 µL of *E. coli* culture were directly added to the tube.

DNA extraction was based on the manufacturer’s protocol. Because the polycarbonate filter is soluble in chloroform, we evaluated whether chloroform extraction would enhance DNA recovery. For this purpose, 400 µL of chloroform were added to the tube before the addition of lysis buffer. With the exception of this chloroform treatment, the procedures were performed in accordance with the manufacturer’s protocol.

*E. coli* 16S rRNA genes were enumerated using primers and TaqMan hydrolysis probes (Suzuki *et al.* 2000). qPCR was performed using a Roche LightCycler 480 System (Roche diagnostics, Germany). qPCR was performed in a reaction volume of 20 µL. Each reaction contained 200 nM of each primer, 100 nM of each probe, and 1× LightCycler 480 Probe Master (Roche). qPCR was conducted by initial denaturation at 95℃ for 10 min; followed by 45 cycles of denaturation at 95℃ for 10 s, annealing at 60℃ for 30 s, and extension at 72℃ for 1 s. 16S rRNA gene copy numbers were normalized to copy numbers of the *E. coli* 16S rRNA gene. The cell number determined by qPCR was normalized to the cell number determined by cell counting; this was regarded as the recovery efficiency.

The obtained DNA recovery efficiencies agree with the similar previous study (Sauer et al. 2011; DNA recovery efficiency were 15.3 ± 2.7 %)

***References***

Honjo M., Matsui K., Ueki M., Nakamura R., Fuhrman J. A. and Kawabata Z. i. 2006 Diversity of virus-like agents killing *Microcystis aeruginosa* in a hyper-eutrophic pond. *Journal of Plankton Research* **28**, 407-412.

Sauer, E.P., VandeWalle, J.L., Bootsma, M.J., McLellan, S.L., 2011. Detection of the human specific Bacteroides genetic marker provides evidence of widespread sewage contamination of stormwater in the urban environment. *Water Research* **45**, 4081-4091.

Stoddard S. F., Smith B. J., Hein R., Roller B. R. K. and Schmidt T. M. 2015 rrnDB: improved tools for interpreting rRNA gene abundance in bacteria and archaea and a new foundation for future development. *Nucleic Acids Research* **43**, D593-D598.

Suzuki M. T., Taylor L. T. and DeLong E. F. 2000 Quantitative analysis of small-subunit rRNA genes in mixed microbial populations via 5 '-nuclease assays. *Applied and Environmental Microbiology* **66**, 4605-4614.

**Table S4** Primers used for screening of tetracycline resistant bacteria

Aarestrup F. M., Agerso Y., Gerner-Smidt P., Madsen M. and Jensen L. B. 2000 Comparison of antimicrobial resistance phenotypes and resistance genes in Enterococcus faecalis and Enterococcus faecium from humans in the community, broilers, and pigs in Denmark. *Diagn Microbiol Infect Dis* **37**, 127-137.

Eden P. A., Schmidt T. M., Blakemore R. P. and Pace N. R. 1991 Phylogenetic analysis of Aquaspirillum magnetotacticum using polymerase chain reaction-amplified 16S rRNA-specific DNA. *Int J Syst Bacteriol* **41**, 324-325.

Huber R., Eder W., Heldwein S., Wanner G., Huber H., Rachel R. and Stetter K. O. 1998 Thermocrinis ruber gen. nov., sp. nov., a Pink-Filament-Forming Hyperthermophilic Bacterium Isolated from Yellowstone National Park. *Applied and Environmental Microbiology* **64**, 3576-3583.

Ng L. K., Martin I., Alfa M. and Mulvey M. 2001 Multiplex PCR for the detection of tetracycline resistant genes. *Molecular and Cellular Probes* **15**, 209-215.

Peak N., Knapp C. W., Yang R. K., Hanfelt M. M., Smith M. S., Aga D. S. and Graham D. W. 2007 Abundance of six tetracycline resistance genes in wastewater lagoons at cattle feedlots with different antibiotic use strategies. *Environmental Microbiology* **9**, 143-151.

Poppe C., Martin L., Muckle A., Archambault M., McEwen S. and Weir E. 2006 Characterization of antimicrobial resistance of Salmonella Newport isolated from animals, the environment, and animal food products in Canada. *Canadian Journal of Veterinary Research-Revue Canadienne De Recherche Veterinaire* **70**, 105-114.

Srinivasan V., Nam H. M., Sawant A. A., Headrick S. I., Nguyen L. T. and Oliver S. P. 2008 Distribution of tetracycline and streptomycin resistance genes and class 1 integrons in Enterobacteriaceae isolated from dairy and nondairy farm soils. *Microbial Ecology* **55**, 184-193.

Tamminen M., Karkman A., Lohmus A., Muziasari W. I., Takasu H., Wada S., Suzuki S. and Virta M. 2011 Tetracycline Resistance Genes Persist at Aquaculture Farms in the Absence of Selection Pressure. *Environmental Science & Technology* **45**, 386-391.

Turner S., Pryer K. M., Miao V. P. and Palmer J. D. 1999 Investigating deep phylogenetic relationships among cyanobacteria and plastids by small subunit rRNA sequence analysis. *J Eukaryot Microbiol* **46**, 327-338.

**Table S5** Primers and probes used in quantitative PCR assays

Knapp C. W., Dolfing J., Ehlert P. A. I. and Graham D. W. 2010 Evidence of increasing antibiotic resistance gene abundances in archived soils since 1940. *Environmental Science & Technology* **44**, 580-587.

Nolvak H., Truu M., Tiirik K., Oopkaup K., Sildvee T., Kaasik A., Mander U. and Truu J. 2013 Dynamics of antibiotic resistance genes and their relationships with system treatment efficiency in a horizontal subsurface flow constructed wetland. *Science of the Total Environment* **461**, 636-644.

Srinivasan V., Nam H. M., Sawant A. A., Headrick S. I., Nguyen L. T. and Oliver S. P. 2008 Distribution of tetracycline and streptomycin resistance genes and class 1 integrons in *Enterobacteriaceae* isolated from dairy and nondairy farm soils. *Microbial Ecology* **55**, 184-193.

**Table S6** Statistics table for Fig. 2

Results for pairwise PERMDISP analysis. The numeric values in the table are P value from each pairwise comparison with the adjusted significance level (=0.01/3). The P values from the overall PERMIDISP analysis were 0.03, 0.02, 0.001, and 0.001 for physicochemical parameters, bacterial abundance and chlorophyll a, microbial functioning, and bacterial community structure, respectively.

**Table S7** Statistics table for Fig. S3

Results for pairwise PERMANOVA analysis coupled with PERMDISP. The numeric values in the table are P value from each pairwise comparison with the adjusted significance level (=0.01/3). The P values from the overall PERMANOVA analysis were 0.001, 0.003, 0.001, and 0.001 for physicochemical parameters, bacterial abundance and chlorophyll a, microbial functioning, and bacterial community structure, respectively.

**Table S7** *(continued)*

Summary table for PERMANOVA corresponding to Fig. S3

Physicochemical parameters (Fig. S3a)

**Factor df SS F #Perm Pr(>F)**

Place 2 1.2079 10.547 999 0.001

Residuals 37 2.1188

Bacterial abundance and chlorophyll a (Fig. S3b)

**Factor df SS F #Perm Pr(>F)**

Place 2 4.3341 7.1342 999 0.003

Residuals 37 11.2389

Microbial functioning (Fig. S3c)

**Factor df SS F #Perm Pr(>F)**

Place 2 1.6140 7.7085 999 0.001

Residuals 37 3.8735

Bacterial community structure (Fig. S3d)

**Factor df SS F #Perm Pr(>F)**

Place 2 5.8489 7.0381 999 0.001

Residuals 37 15.3740

**Table S8** Identified tetracycline resistant environmental isolates carrying *tet* genes

*Cp* (Cycle number)

Log concentration (gene copies per reaction)

**Fig. S1** Standard curve of *tet*(A), *tet*(B) and *tet*(M) for qPCR assay. Each qPCR assay include standard curve with plotting crossing point (*Cp*) value vs. log10 values of the gene copy number. *Cp* value was determined by second derivative method. A limit of quantification was defined as the lowest gene copy number (7.5 copies per reaction) within the linear range of quantification.


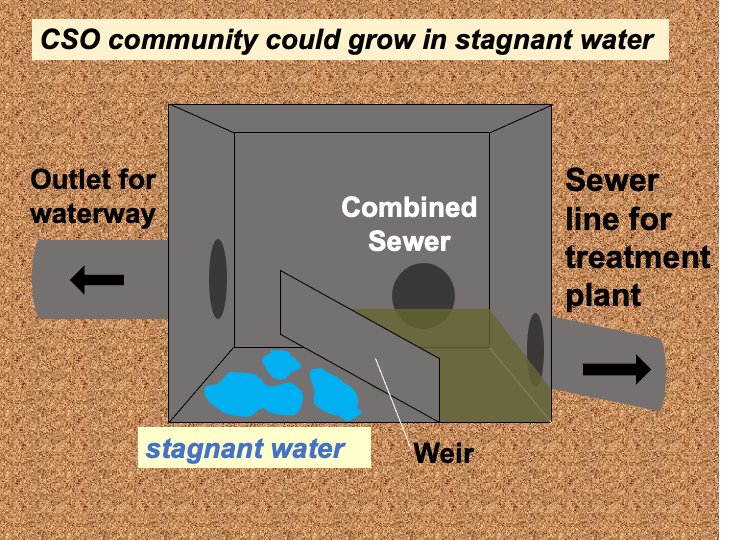


**c**

**Fig. S2** Depicted image of stagnant water in combined sewer overflow structure. The largest structure had a weir measuring 3900 mm long × 550mm

high. There are 28 release outlets on the H- waterway and D-waterway.


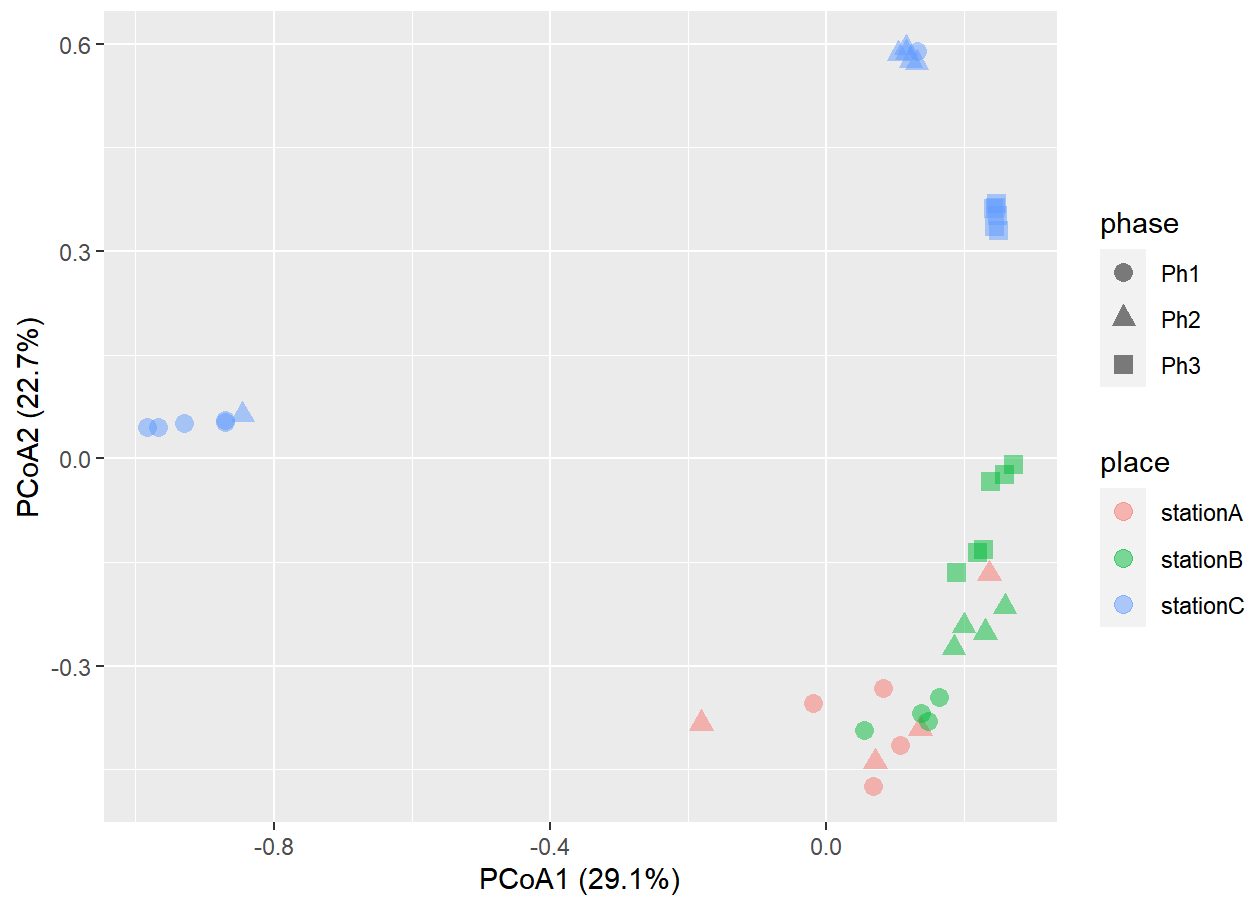

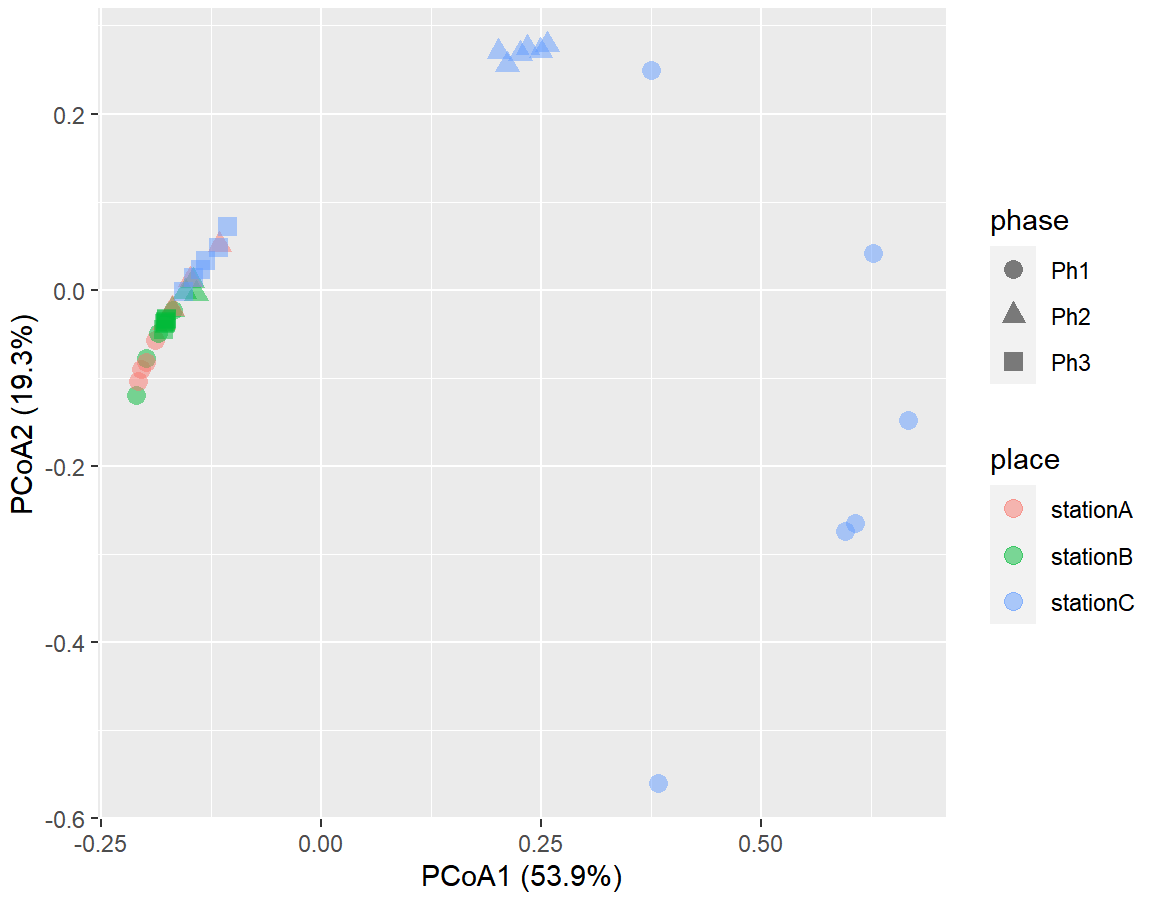

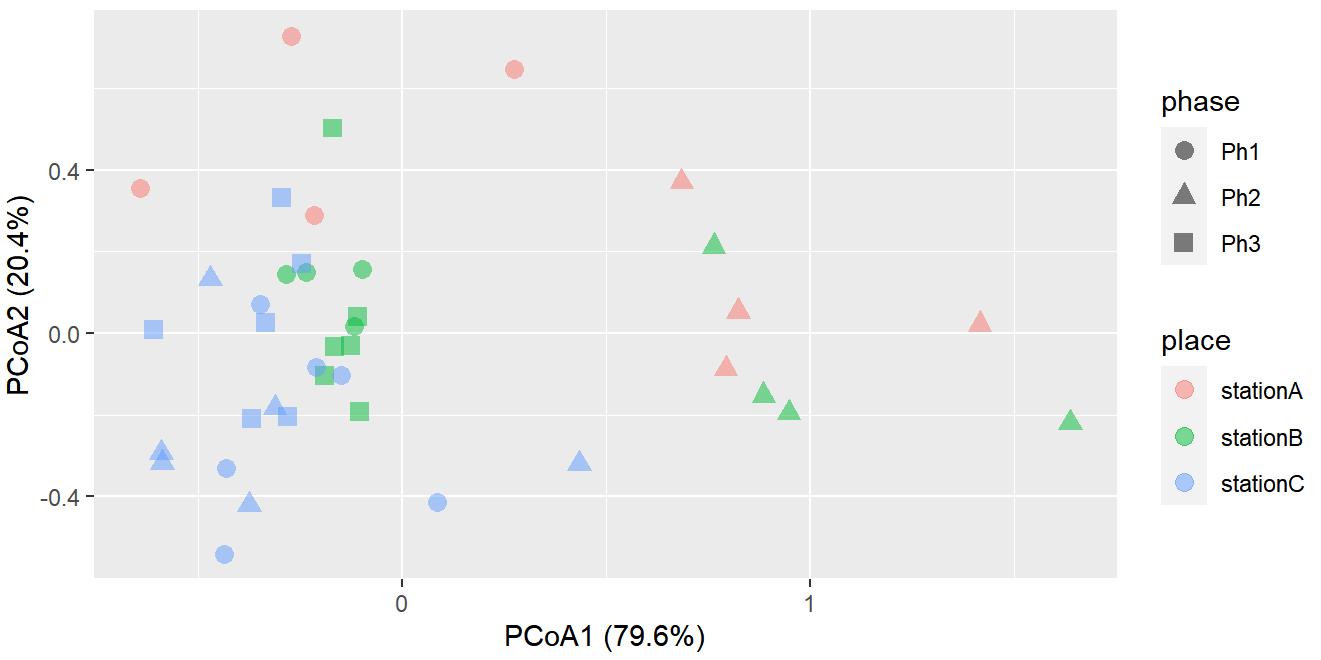

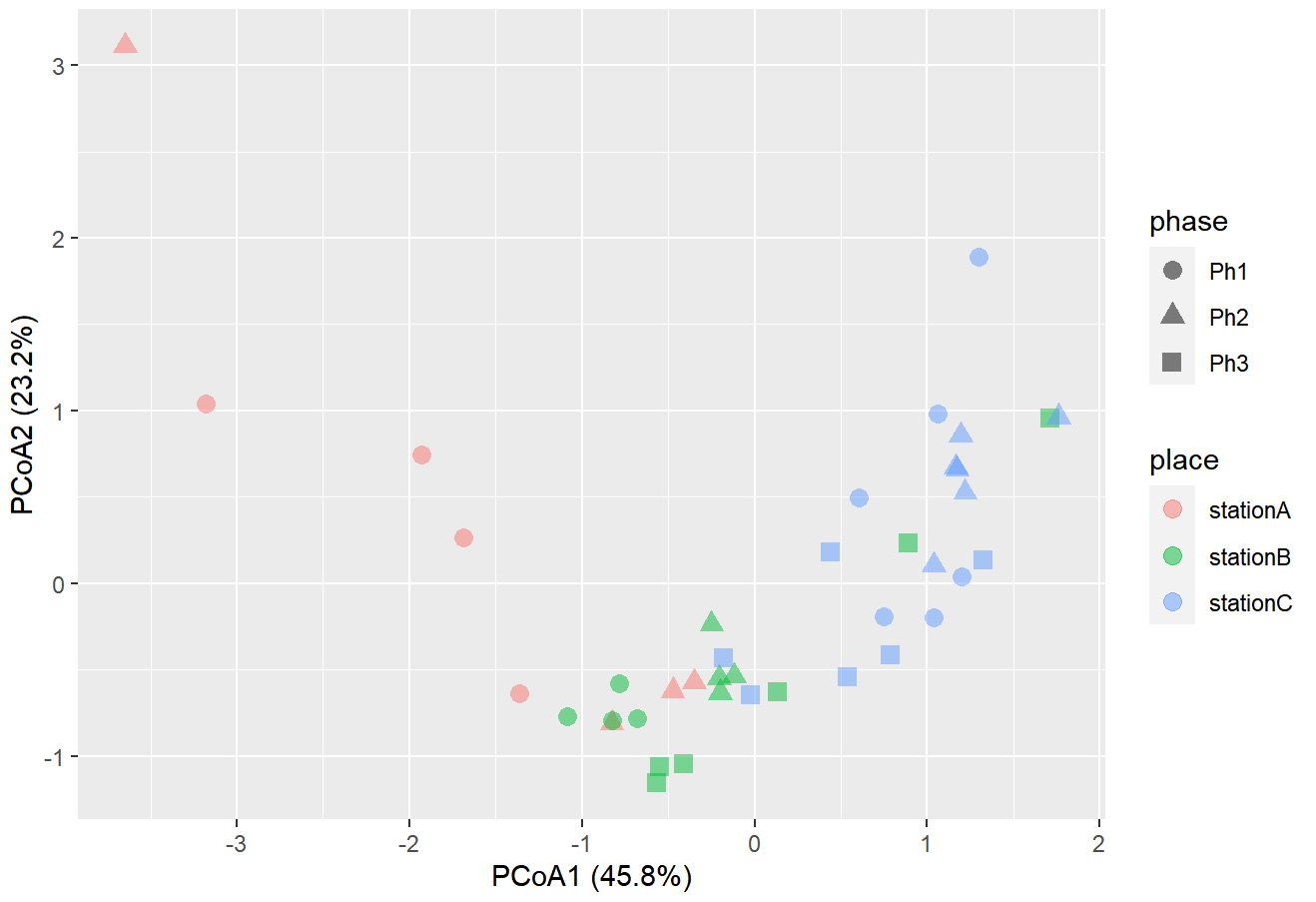


**Fig. S3** Principal Coordinate Analysis (PCoA) of physicochemical and ecological variables from 3 stations sampled in 3 phases. Points indicate a) physicochemical parameters, b) bacterial abundance and chlorophyll a, c) bacterial community functions, and d) bacterial community composition samples, color denotes the stations, and shape indicates the phases.

**a**

**b**

**c**

**d**


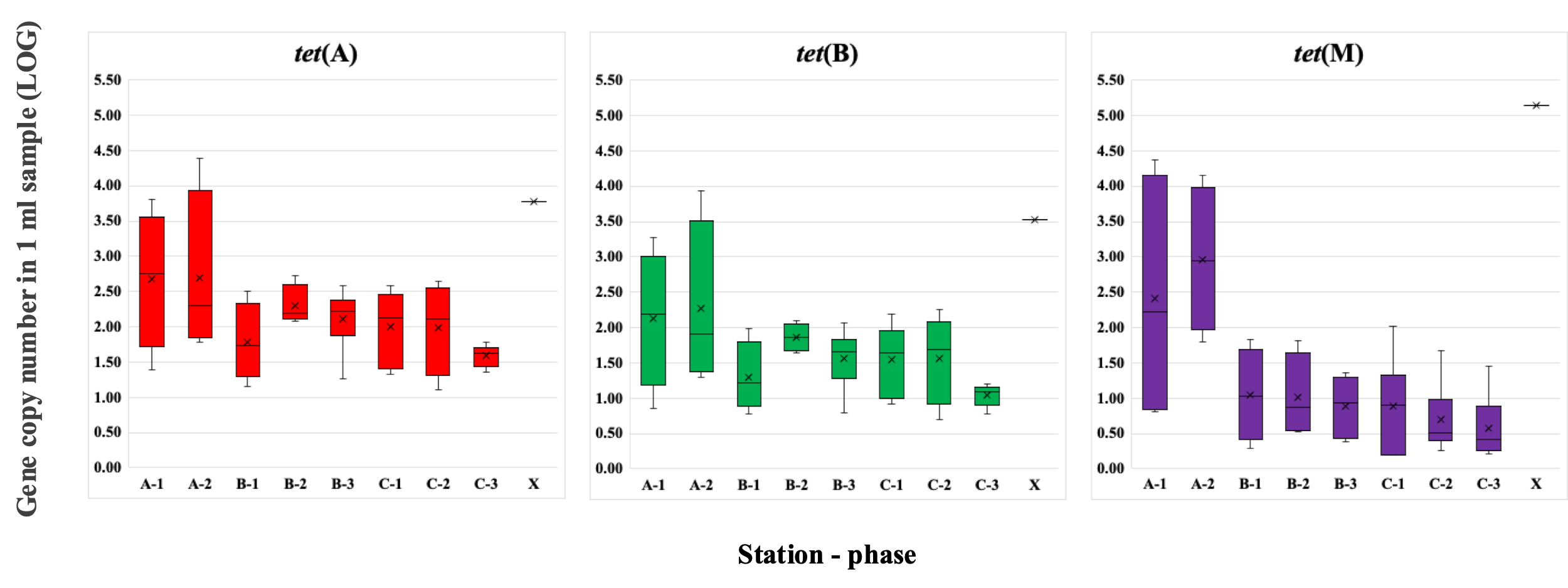


**Fig. S4** The box-and-whisker plots *tet*(A), *tet*(B) and *tet*(M) genes at stations A, B and C in 3 different phases. X denotes the *tet* genes from grab sewer sample.
